# Chinese herbal medicine for patients living with HIV in Guangxi province, China: A propensity score matching analysis of real-world data

**DOI:** 10.1371/journal.pone.0304332

**Published:** 2024-09-06

**Authors:** Jing Li, Chen Shen, Zhen-Wei Liu, Feng-Lan Pu, Shi-Huan Cao, Yue Zhang, Xiao-Feng Han, Peng Yuan, Sheng-Lin Duan, Feng Jiang, Jian-Ping Liu

**Affiliations:** 1 Centre for Evidence-Based Chinese Medicine, Beijing University of Chinese Medicine, Beijing, China; 2 Beijing Key Laboratory of the Innovative Development of Functional Staple and the Nutritional Intervention for Chronic Disease, China National Research Institute of Food & Fermentation Industries Co,. Ltd, Beijing, China; 3 AIDS Centre, Ruikang Hospital, Guangxi University of Traditional Chinese Medicine, Nanning, Guangxi Zhuang Autonomous Region, China; 4 Center for Disease Control and Prevention of Minhang District, Shanghai, China; Icahn School of Medicine at Mount Sinai Department of Pharmacological Sciences, UNITED STATES

## Abstract

**Background:**

From 2004 onwards, the Chinese government has freely offered complimentary Chinese herbal medicine (CHM) to Chinese HIV/AIDS patients, alongside the prescribed first line therapy of highly active antiretroviral therapy (HAART). Thus, we aimed to explore the effectiveness and safety of CHM for patients with HIV/AIDS.

**Methods:**

The data from the Guangxi pilot database and antiviral treatment sites database have been respectively developed into two datasets in this prospective cohort real-world study, the CHM combined HAART group (the integrated group) and the HAART group. A 1:1 propensity score matching (PSM) was performed and the longitudinal data were analyzed using a generalized estimating equation (GEE) model with an autocorrelation matrix and log link function attached to the Gamma distribution.

**Results:**

A final sample of 629 patients, 455 and 174 in the integrated group and HAART group respectively, were obtained from the full dataset. As covariates for PSM, gender, age, baseline CD_4_^+^ and CD_4_^+^/ CD_8_^+^ were assessed based on the results of the logistic regression analyses. Following PSM, 166 pairs from the full dataset were matched successfully, with 98 pairs in the baseline CD_4_^+^ > 200 subgroup, and 55 pairs in the baseline CD_4_^+^ ≤ 200 subgroup. In the full dataset, HAART group achieved higher CD4^+^ count (*OR* = 1.119, 95%*CI* [1.018, 1.230]) and CD4^+^/CD8^+^ ratio (*OR* = 1.168, 95%*CI* [1.045, 1.305]) than the integrated group, so did in the CD4^+^ > 200 subgroup. For the CD_4_^+^ ≤ 200 subgroup, the CD4^+^ (*OR* = 0.825, 95%*CI* [0.694, 0.980]) and CD4^+^/CD8^+^ (*OR* = 0.826, 95%*CI* [0.684, 0.997]) of the integrated group were higher than those of the HAART group. The safety outcomes showed that there were no significant differences in BUN, ALT and AST levels between the groups but Cr showed significantly higher levels in HAART groups of all three datasets.

**Conclusions:**

Compared to HAART alone, CHMs combined with HAART had better effects in improving the immune function of HIV/AIDS in patients with baseline CD_4_^+^ count ≤ 200. The results of the two subgroups are in opposite directions, and chance does not explain the apparent subgroup effect. A study with larger sample size and longer follow-up period is warranted in order to increase study credibility.

## Background

Acquired immunodeficiency syndrome (AIDS) is a chronic and fatal infectious disease caused by the human immunodeficiency virus (HIV). HIV destroys the white blood cells called CD4^+^ cells, weakening a person’s immunity against opportunistic infections, such as tuberculosis and fungal infections, severe bacterial infections and some cancers [1]. According to the Joint United Nations Programme on HIV/AIDS (UNAIDS), HIV/AIDS has driven 37.7 million people to be affected by the end of 2020, with 1.5 million newly HIV infected in that year. About 27.5 million people are now receiving antiretroviral therapy. which is indicated in cases of HIV [2]. Currently, there are six classes of AIDS agents in the world, namely nucleoside reverse transcriptase inhibitors (NRTIs), non-nucleoside reverse transcriptase inhibitors (NNRTIs), protease inhibitors (PIs), integrase inhibitors (INSTIs), fusion inhibitors (FIs) and CCR5 inhibitors [3]. Long-term use of NRTI agents could be attributed to producing adverse reactions of hyperlactatemia and lactic acidosis, neuropathy, pancreatitis and lipoatrophy [4]^.^ The most commonly used NNRTIs, efavirenz is known to have a significant side-effect profile that includes neuropsychiatric toxicity, rash, hyperlipidemia and elevated transaminases [5, 6]. According to the world health organization’s HIV Drug Resistance Report in 2021, more and more nations were reaching the 10% threshold of pretreatment HIV drug resistance (HIVDR) to NNRTIs and it was found up to 3 times more likely in people who had previous exposure to antiretroviral agents [7]. PIs may likewise elicit side effects encompassing metabolic abnormalities including dyslipidemia (primarily triglycerides), insulin resistance, hyperglycemia, and lipodystrophy [8]^.^

Chinese herbal medicine (CHM) was first used to treat HIV-infected people in 1987, when traditional Chinese medicine (TCM) practitioners from China provided medical assistance in Tanzania, Africa [9]. Given its promising effectiveness and high safety profile, CHM has been used in the treatment of HIV/AIDS for more than 30 years as an alternative and complementary therapy to highly active antiretroviral therapy (HAART) [10]. The application of TCM has also been proven to significantly improve patients’ clinical symptoms and signs, improve their working capacity and quality of life, safeguard their immune system, and postpone the onset of AIDS [11–14]. Additionally, the synergistic administration of CHM and antiviral agents can reduce some adverse reactions to antiviral agents [11]. A pilot project in China, the National Free CHM HIV/AIDS Treatment Program (NFCHMP), was launched in 2004 and extended rapidly. NFCHMP was supported by the State Administration of Traditional Chinese Medicine (SATCHM) and the Ministry of Finance, and the program has provided free TCM treatment to tens of thousands of HIV/AIDS patients.

It is apparent that real-world studies (RWSs) are not constrained by the small sample size or the strict inclusion criteria, such as the exclusion of children or the elderly, as are randomized controlled trials (RCTs). As a result, it can contribute to a broad evaluation of treatment modes and external effectiveness [15]. A number of RWSs have been conducted on HIV. For instance, a multicentral RWS by Santinelli et al. assessed the real-life effectiveness, tolerability, and safety of long-term Raltegravir use in elderly HIV infected patients [16]. Similarly, Okoli et al described the actual use and effectiveness of Dolutegravir-based regimens in HIV patients treated in the United Kingdom [17]. A retrospective cohort study was conducted in Henan province on basis of the NFCHMP database and it demonstrated that CHM could decrease the disease progression, reduce the mortality of people living with HIV, and improve life expectancy. However, the predominant limitation was that they chose the contemporaneous world mortality rate as a comparison [18]. A 7-year observational study indicated long-term utilisation of CHM could keep up or impede the pace of CD4^+^ cell counts declining. However, this study did not address bias, potential confounders and the possibility that results may have occurred by chance [19]. RWSs employing TCM to treat HIV are, whereas, limited in small sample size, do not address confounding factors, or ignored individual disease progression.

RWS, on the other hand, may be accompanied by more confounding factors than RCTs. Thus, propensity score-based approaches have been developed to reduce or remove the factors [20]. The propensity score represents the probability of assigning treatment conditions on observed baseline attributes. Furthermore, Liang and Zeger proposed the generalized estimating equation (GEE) to analyze real-world data (RWD), which was developed just on the basis of the generalized linear model (GLM) and enhance GLM to accommodate the modelling of correlated data. GEE is appropriate for complete data or missing data at random [21]. Therefore, we aimed to analyze the longitudinal data using propensity score matching (PSM) and GEE to explore the effectiveness and safety of CHM for patients with HIV/AIDS.

## Methods

### Ethics approval and consent to participate

This study was approved by the ethics committee of the Beijing University of Chinese Medicine (BZZYYDX-LL20160215).

### Data source

The ethics committee of the Beijing University of Chinese Medicine approved this study before data collection began (BZZYYDX-LL20160215). The prospective cohort study was based on two registration databases, the Guangxi pilot database of the NFCHMP (hereinafter referred to as Guangxi pilot database) and the antiviral treatment site database of Ruikang Hospital affiliated with the Guangxi University of Traditional Chinese Medicine (hereinafter referred to as antiviral treatment sites database). The study participants provided their written informed consent and permitted the use of their medical information.

### Participants

The real-world data was composed of two sets, namely the CHM combined with the HAART group (integrated group for short) and the HAART group. The participants in the integrated group were sourced from the Guangxi pilot database and those in the HAART group were sourced from the antiviral treatment sites database. Over the course of 36 months, all participants were followed up every three months.

Eligible participants were those diagnosed with HIV/AIDS and receiving HAART treatment between 2004 and 2016. A complete set of included case data should be provided with all necessary information. Participants were excluded if they did not have baseline characteristics (gender, age, marital status, possible route of infection) or CD4^+^ baseline data. In the case of participants with all follow-up data missing within 36 months, they were excluded from the study.

### Exposure factors

This study was divided into the integrated group and the HAART group based on whether participants received CHMs treatment or not. There was no limit to the duration of the exposure. CHMs were available in three forms: Tangcao Tablets, containing *Geranium wilfordii* Maxim., *Lonicera japonica* Thunb., *Trichosanthes kirilowii* Maxim., *Bupleuri Radix*, *Elsholtzia ciliata*, *Punica granatum* L., *Astragalus membranaceus* (Fisch.) Bunge, *Glycyrrhizae Radix* et Rhizoma, *Bombax ceiba* L., *Millettia dielsiana*, *Carthamus tinctorius* L., *Oryza sativa* L., *Terminalia chebula* Retz., *Scleromitrion diffusum* (Willd.) R.J. Wang, *Trapa bispinosa* Roxb., *Ginkgo biloba* L., *Portulaca oleracea* L., *Neopicrorhiza scrophulariiflora* (Pennell) D. Y. Hong, *Solanum nigrum* L. and *Buthus martensii* Karsch; Qingdu Capsules/Granules, mainly composed of *Astragalus membranaceus* (Fisch.) Bunge, *Atractylodes Lancea* (Thunb.) DC., *Polyrhachis vicina* Roger, *Scutellaria baicalensis* Georgi, *Poria cocos* (Schw.) Wolf, *Ganoderma lucidum* (Curtis) P. Karst., Andrographis paniculata (Burm. f.) Wall. ex Nees in Wallich and *Gynostemma pentaphyllum* (Thunb.) Makino, and Shenling Fuzheng Capsules, mainly composed of *Codonopsis pilosula* (Franch.) Nannf., *Astragalus membranaceus* (Fisch.) Bunge, *Atractylodes macrocephala* Koidz., *Gynostemma pentaphyllum* (Thunb.) Makino, *Polyrhachis vicina* Roger and *Ganoderma lucidum* (Curtis) P. Karst. A HAART regimen was administered in accordance with the *National Free Antiretroviral Treatment Manual Book*. The free drugs used in 2004 included domestic drugs Zidovudine, Stavudine, Didanosine, Nevirapine, and imported drugs Lamivudine, Efavirenz, and Indinavir. As of 2012, Tenofovir was included among the first-line drugs.

### Outcomes

Over a period of 36 months, the primary outcome was CD_4_^+^ T cell count. Secondary outcomes were CD_8_^+^ T cell count, CD_4_^+^/ CD_8_^+^ ratio, the level of creatinine (Cr), blood urea nitrogen (BUN), alanine transaminase (ALT) and aspartate aminotransferase (AST).

### Data analysis

Data processing and data analysis were undertaken using Excel 2016 and SPSS 22.0, respectively. For normally distributed quantitative data (CD4^+^ and CD8^+^), we used the Pauta criterion (values beyond $\bar{X}$ ± 3S) to detect outliers. After verification, outliers and anomalies that represent accurate values were still included in the analysis. The continuous variables were statistically described by means and standard deviation, median and 95% confidence intervals (CIs), and were compared between groups using a t-test or Wilcoxon rank-sum test. Categorical variables were described by constituent ratios, and intergroup comparisons were conducted using the chi-square test or Fisher exact probability method. To explore the covariates associated with PSM, a logistic regression model was applied. The PSM was carried out using the R plug-in in SPSS. In the GEE model, an autocorrelation matrix of an autoregressive AR (1) process was selected in the study for dealing with data at different time points. Statistically significant was determined by P < 0.05.

In the logistic regression model, the outcome variable was the CD_4_^+^ cell count change. Using the relative magnitude of the difference between the CD_4_^+^ cell count at the last follow-up visit and baseline for each patient, and the mean of these differences for all patients, the change in CD_4_^+^ cell count has been expressed. In cases where the difference was greater than the mean of the differences (dominant population), 1 was used, while in cases where the difference was less than the mean of the differences (inferior population), 0 was used. Let a binary dependent variable be set as Y, then:

Y={1,dominantpopulation(positiveresult)0,inferiorpopulation(negativeresult)

As possible covariates of PSM, the following variables may affect the immune function of HIV/ AIDS patients: gender, age, marital status, possible route of infection, baseline CD_4_^+^ count, baseline CD_8_^+^ count, baseline CD_4_^+^ / CD_8_^+^ T-cell ratio, and baseline level of Cr, BUN, ALT and AST. Upon considering the 11 independent variables, a logistic regression model [22], was used to calculate the probability of obtaining positive results.

A propensity score (PS) is defined as the conditional probability of assigning a research object to the treatment group when multiple covariates are present [23]. The baseline characteristic variables selected in this study were used as the matching factors, and the 1:1 matching was performed based on the principles of nearest neighbour matching and calliper matching (calliper value: 0.03) [24]. In addition, the value of CD_4_^+^≤ 200 was of important clinical significance and was considered to be in the AIDS stage. Hence, the PSM was conducted within two subgroups respectively, baseline CD_4_^+^ count > 200 and baseline CD_4_^+^ count ≤ 200.

## Results

### Sample characteristics

**Fig 1:**
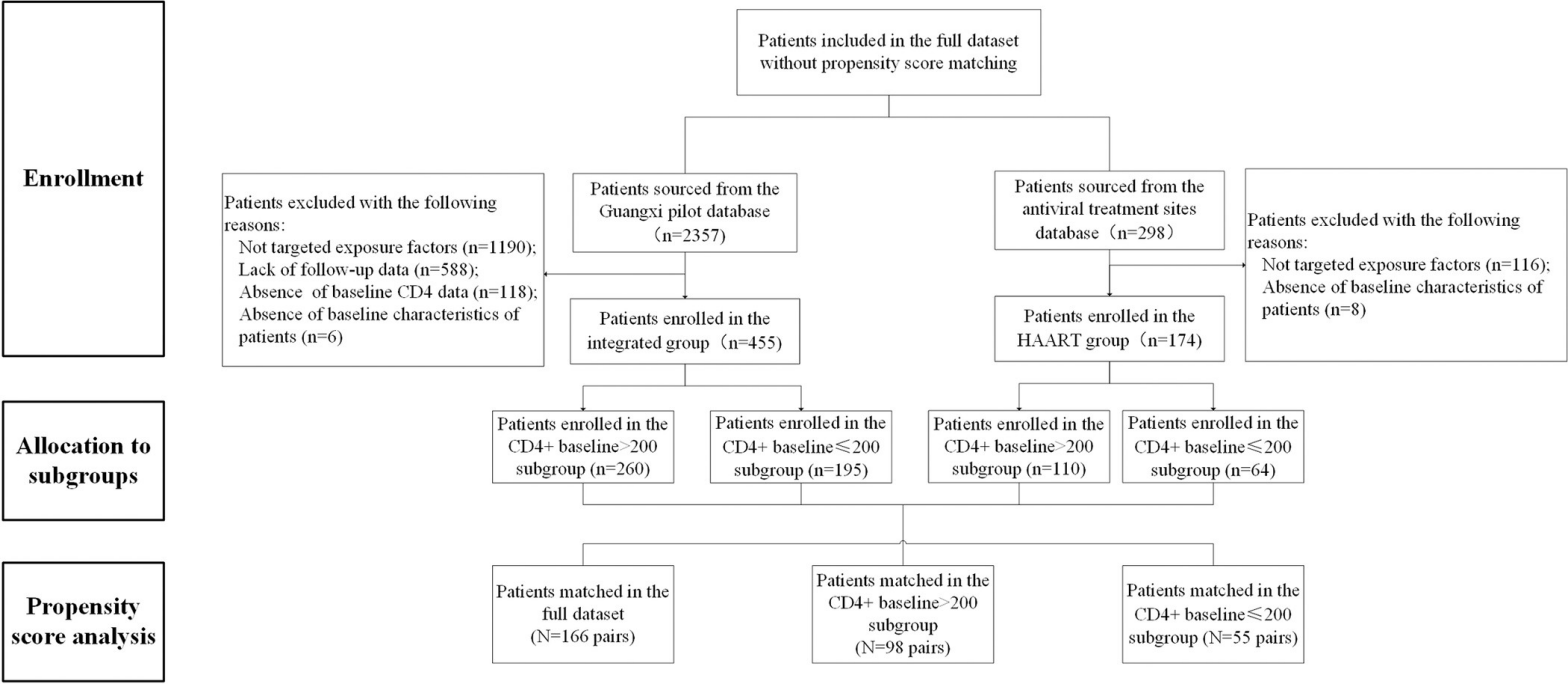
Patients flow diagram for the prospective cohort study.

The identification and selection process of the study sample is shown in Fig 1. A final sample of 629 patients, 455 in the integrated group and 174 in the HAART group, was obtained. Baseline characteristics of the samples by groups, without PSM or with PSM, are shown in Table 1. The results of single-factor and multifactor logistic regression analysis were represented in S1 and S2 Tables, Taking into account the results of the logistic regression analyses and important clinical indicators, gender, age, baseline CD_4_^+^ count and CD_4_^+^/ CD_8_^+^ were incorporated as covariates for the PSM. PSM enabled matching of 166 pairs across the full dataset were matched successfully, along with 98 pairs in the subgroup with a baseline CD_4_^+^ count > 200, and 55 pairs in the subgroup with a baseline CD_4_^+^ count≤ 200.

**Table 1:** Sample characteristics at baseline by groups.

Variables	Full dataset (without matching)			Full dataset (matched)			CD_4_^+^ baseline> 200 (without matching)			CD_4_^+^ baseline> 200 (matched)			CD_4_^+^ baseline≤ 200 (without matching)			CD_4_^+^ baseline≤ 200 (matched)		
	Integrated group (*n* = 455)	HAART group (*n* = 174)	*P* value	Integrated group (*n* = 166)	HAART group (*n* = 166)	*P* value	Integrated group (*n* = 260)	HAART group (*n* = 110)	*P* value	Integrated group (*n* = 88)	HAART group (*n* = 88)	*P* value	Integrated group (*n* = 195)	HAART group (n = 64)	*P* value	Integrated group (*n* = 55)	HAART group (*n* = 55)	*P* value
**Gender—n (%)**																		
n	455	174	0.32	166	166	1.00	260	110	0.98	98	98	1.00	195	64	0.15	55	55	0.66
Male	322 (70.77%)	116 (66.67%)		112 (67.47%)	112 (67.47%)		165 (63.46%)	70 (63.64%)		62 (63.27%)	62 (63.27%)		157 (80.51%)	46 (71.88%)		40 (72.73%)	42 (76.36%)	
Female	133 (29.23%)	58 (33.33%)		54 (32.53%)	54 (32.53%)		95 (36.54%)	40 (36.36%)		36 (36.73%)	36 (36.73%)		38 (19.49%)	18 (28.12%)		15 (27.27%)	13 (23.64%)	
**Age—year**																		
n	455	174	<0.01	166	166	0.42	260	110	0.04	98	98	0.59	195	64	0.02	55	55	0.47
Median	44	38		41	38		42	38.5		39	39		46	37.5		44	38	
IQR	35, 57	30, 55		33, 51	31, 55.25		35, 54	29, 55		34, 52	29, 56.25		36, 58	31, 54.25		33, 57	32, 57	
**Marital Status—n (%)**																		
n	455	174	0.72	166	166	0.99	260	110	0.83	98	98	0.98	195	64	0.86	55	55	0.84
Unmarried	68 (14.95%)	32 (18.39%)		31 (18.67%)	31 (18.67%)		40 (15.38%)	20 (18.18%)		15 (15.31%)	16 (16.33%)		28 (14.36%)	12 (18.75%)		8 (14.55%)	10 (18.18%)	
Married	300 (65.93%)	111 (63.79%)		106 (63.86%)	104 (62.65%)		174 (66.92%)	71 (64.55%)		64 (65.31%)	63 (64.29%)		126 (64.61%)	40 (62.50%)		36 (65.45%)	37 (67.27%)	
Divorced	26 (5.72%)	8 (4.60%)		8 (4.82%)	8 (4.82%)		16 (6.15%)	5 (4.55%)		6 (6.12%)	5 (5.10%)		10 (5.13%)	3 (4.69%)		2 (3.64%)	2 (3.64%)	
Death of a spouse	61 (13.40%)	23 (13.22%)		21 (12.65%)	23 (13.86%)		30 (11.55%)	14 (12.72%)		13 (13.26%)	14 (14.28)		31 (15.90%)	9 (14.06%)		9 (16.36%)	6 (10.91%)	
**Possible route of infection—n (%)**																		
n	455	174	<0.01	166	166	<0.01	260	110	<0.01	98	98	0.03	195	64	<0.01	55	55	0.09
Drug-taking	52 (11.42%)	11 (6.32%)		24 (14.46%)	11 (6.63%)		33 (12.69%)	7 (6.36%)		12 (12.25%)	7 (7.14%)		19 (9.74%)	4 (6.25%)		6 (10.90%)	4 (7.30%)	
Sexual contact	383 (84.18%)	145 (83.33%)		135 (81.33%)	138 (83.13%)		214 (82.31%)	90 (81.82%)		80 (81.63%)	79 (80.61%)		169 (86.67%)	55 (85.94%)		48 (87.30%)	46 (83.60%)	
Mother-to-child transmission	3 (0.66%)	17 (9.77%)		1 (0.60%)	16 (9.64%)		2 (0.77%)	12 (10.91%)		2 (2.04%)	11 (11.22%)		1 (0.51%)	5 (7.81%)		0 (0)	5 (9.10%)	
Paid blood	2 (0.44%)	0 (0)		2 (1.20%)	0 (0)		0 (0)	0 (0)		0 (0)	0 (0)		2 (1.03%)	0 (0)		0 (0)	0 (0)	
Unknown	15 (3.30%)	1 (0.58%)		4 (2.41%)	1 (0.60%)		11 (4.23%)	1 (0.91%)		4 (4.08%)	1 (1.02%)		4 (2.05%)	0 (0)		1 (1.80%)	0 (0)	
**CD**_**4**_^**+**^ **—cell/ul**																		
n	455	174	0.23	166	166	0.10	260	110	0.42	98	98	0.11	195	64	0.18	55	55	0.99
Median	227	243.5		201	235.50		282	312		262.5	307.5		133	126		117	126	
IQR	148, 295	154.75, 344.25		132.5, 282	150.5, 339.5		240, 388.25	248.75, 372.25		231.75, 377	246.5, 370.25		92, 172	66.5, 170.75		75, 175	66, 175	
**CD**_**8**_^**+**^ **—cell/ul**																		
n	452	172	<0.01	164	165	0.33	259	109	<0.01	98	98	0.50	193	63	<0.01	54	54	0.82
Median	731	921.5		858	895		831.00	956.00		938	952		655	840		816.5	772.50	
IQR	508, 1051.75	665, 1270		569, 1189.75	652, 1182.5		545, 1174	704.5, 1370		645, 1188	700, 1261		467.5, 932.5	560, 1176		532.75, 1024.25	555.75, 1079.75	
**CD**_**4**_^**+**^ **/CD**_**8**_^**+**^																		
n	452	172	<0.01	164	165	0.87	259	109	<0.01	98	98	0.77	193	63	<0.01	54	54	0.63
Median	0.3	0.25		0.24	0.25		0.41	0.33		0.34	0.33		0.19	0.12		0.16	0.14	
IQR	0.19, 0.47	0.15, 0.39		0.15, 0.37	0.16, 0.4		0.27, 0.57	0.23, 0.45		0.24, 0.47	0.24, 0.44		0.12, 0.3	0.08, 0.22		0.09, 0.21	0.08, 0.22	
**Cr—μmol/L**																		
n	416	147	<0.01	156	141	<0.01	236	95	<0.01	89	85	<0.01	180	52	<0.01	48	48	<0.01
Median	72	84		72	85		70.95	84		71	84		72.55	84		69.5	85.5	
IQR	62.3, 83.75	72, 97		62.48, 82.8	72, 97		59.38, 82	72, 97		60.5, 81	72, 95		64.3, 86.98	69, 97		63.23, 81.03	73.5, 97	
**BUN—mmol/L**																		
n	431	147	0.01	159	141	0.46	246	95	0.24	94	85	0.90	185	52	<0.01	49	48	0.09
Median	4.66	4.31		4.33	4.31		4.67	4.4		4.49	4.4		4.66	4.13		4.5	4.2	
IQR	3.68, 5.7	3.57, 5.07		3.55, 5.3	3.57, 5.10		3.64, 5.64	3.66, 5.11		3.59, 5.5	3.64, 5.19		3.72, 5.77	3.2, 4.90		3.72, 5.77	3.23, 5.03	
**ALT—U/L**																		
n	434	172	0.09	160	164	0.07	248	109	0.01	95	97	0.11	186	63	0.74	49	54	0.87
Median	21.55	19		21.5	19		22	19		22.4	18		20.85	22		23	23.5	
IQR	15.4, 34	13.25, 32		15.78, 34	13, 32		16, 35	13, 31		15.8, 32	13, 31.5		15, 32.2	15, 35		13.8, 38	15, 37	
**AST—U/L**																		
n	430	172	0.65	158	164	0.77	246	109	0.52	94	97	0.69	184	63	0.08	47	54	0.24
Median	24	25		24	24.5		24	23		24	23		24.05	26		26	27	
IQR	19.7, 34.48	20, 34.75		18.9, 34.08	20, 33.75		19.7, 35.03	19, 31		20, 33.2	19, 31		19.53, 34	21, 41		20, 35	21, 41	

Notes: Cr refers to creatinine; BUN refers to blood urea nitrogen; ALT refers to alanine transaminase; AST refers to aspartate aminotransferase

### CD4+ cell count

The results of CD_4_^+^ cell count over time in patients before PSM are presented in S3 Table. After PSM, Table 2 illustrates the changes in CD_4_^+^ among AIDS patients based on their treatment every three months during the follow-up period. In the full dataset, a significant difference was observed between the groups at 9, 15, 18, 30 and 33 months of follow-up and the CD_4_^+^ levels were all higher in the WM group than that was in the integrated group. According to the statistical significance in the baseline CD_4_^+^ > 200 subgroup, detected from 3 to 33 months of follow-up, the CD_4_^+^ levels in the HAART group were all higher than that in the integrated group. In the subgroup with baseline CD_4_^+^ ≤ 200, CD_4_^+^ was significantly different at the 3rd, 9th and 15th-month follow-up, with higher levels in the integrated group.

**Table 2:** CD_4_^+^ cell count in HIV/AIDS patients (Full dataset, CD_4_^+^ baseline > 200, baseline CD_4_^+^≤ 200) by treatment during follow-up after PSM.

Variable	Full dataset			CD_4_^+^ baseline > 200			CD_4_^+^ baseline ≤ 200		
	Integrated group (n = 166)	HAART group (n = 166)	P values	Integrated group (n = 98)	HAART group (n = 98)	P values	Integrated group (n = 55)	HAART group (n = 55)	P values
**CD**_**4**_^**+**^ **(baseline)—cell/ul**									
n	166	166	0.10	98	98	0.11	55	55	0.99
Median	201	235.5		262.50	307.5		117	126	
IQR	132.5, 282	150.5, 339.5		231.75, 377	246.5, 370.25		75, 175	66, 175	
**CD**_**4**_^**+**^ **(3 months)—cell/ul**									
n	58	147	0.53	27	91	0.04	16	45	0.01
Median	409.5	390		371	457		448.5	248	
IQR	251.75, 534.25	253, 537		269, 508	351, 589		249.5, 621.5	170.5, 344.5	
**CD**_**4**_^**+**^ **(6 months)—cell/ul**									
n	51	145	0.34	26	88	0.01	10	47	0.07
Median	418	380		348.5	451		461	276	
IQR	274, 592	280.5, 510		224.75, 560.25	374.75, 563.25		206.25, 729.5	194, 330	
**CD**_**4**_^**+**^ **(9 months)—cell/ul**									
n	49	123	0.02	34	75	<0.01	11	37	0.01
Median	329	418		328.5	458		427	254	
IQR	216, 477.5	291, 535		216, 465	378, 581		223, 625	205.5, 391	
**CD**_**4**_^**+**^ **(12 months)—cell/ul**									
n	41	125	0.41	25	73	<0.01	15	38	0.13
Median	397	424		363	467		397	271.5	
IQR	208, 570.5	276.5, 525.5		205.5, 491.5	396.5, 586.5		205, 554	212.5, 363.25	
**CD**_**4**_^**+**^ **(15 months)—cell/ul**									
n	58	91	0.04	35	58	<0.01	22	24	0.01
Median	402	453		330	499		435.5	280	
IQR	210.25, 546.75	334, 577		194, 528	437.25, 644.25		324.75, 567.25	218.25, 378.5	
**CD**_**4**_^**+**^ **(18 months)—cell/ul**									
n	45	85	<0.01	20	50	<0.01	16	22	0.18
Median	314	458		328	499.5		275	338.5	
IQR	206, 568.5	348, 609.5		243.75, 478.5	435.25, 656.75		142.25, 462	257.75, 455	
**CD**_**4**_^**+**^ **(21 months)—cell/ul**									
n	38	69	0.07	36	42	<0.01	15	20	0.86
Median	392.5	457		393	511		334	325	
IQR	260.75, 528.5	346, 544		234, 522.25	427.5, 599.5		225, 709	296.5, 406.25	
**CD**_**4**_^**+**^ **(24 months)—cell/ul**									
n	35	52	0.13	24	28	<0.01	11	14	0.85
Median	398	478.5		408.5	556.5		405	322.5	
IQR	242, 559	314.25, 608.5		264.75, 464.25	379.75, 653.75		193, 623	254.75, 501	
**CD**_**4**_^**+**^ **(27 months)—cell/ul**									
n	48	38	0.19	34	22	<0.01	17	12	0.59
Median	415.5	446.50		311.5	525.5		265	370	
IQR	248.75, 577	355.25, 610.75		204.25, 487.25	445.5, 636.5		162.5, 525.5	263.75, 412	
**CD**_**4**_^**+**^ **(30 months)—cell/ul**									
n	42	20	0.02	28	13	<0.01	17	4	0.76
Median	349.5	431.5		377.5	479		312	326	
IQR	259.5, 447.25	369.75, 549		286.25, 441.75	383.5, 563.5		205, 504	252.25, 633	
**CD**_**4**_^**+**^ **(33 months)—cell/ul**									
n	47	13	<0.01	20	7	<0.01	19	4	0.67
Median	331	451		259	587		365	399.5	
IQR	221, 558	383.5, 643		121.25, 411	387, 855		155, 566	359.75, 443	
**CD**_**4**_^**+**^ **(36 months)—cell/ul**									
n	23	12	0.88	18	6	0.09	6	4	0.35
Median	475	462		403	488		260	423.5	
IQR	244, 701	409, 499.25		235.5, 480.25	412.25, 701.5		172.75, 553	378.25, 481.5	

**Fig 2:**
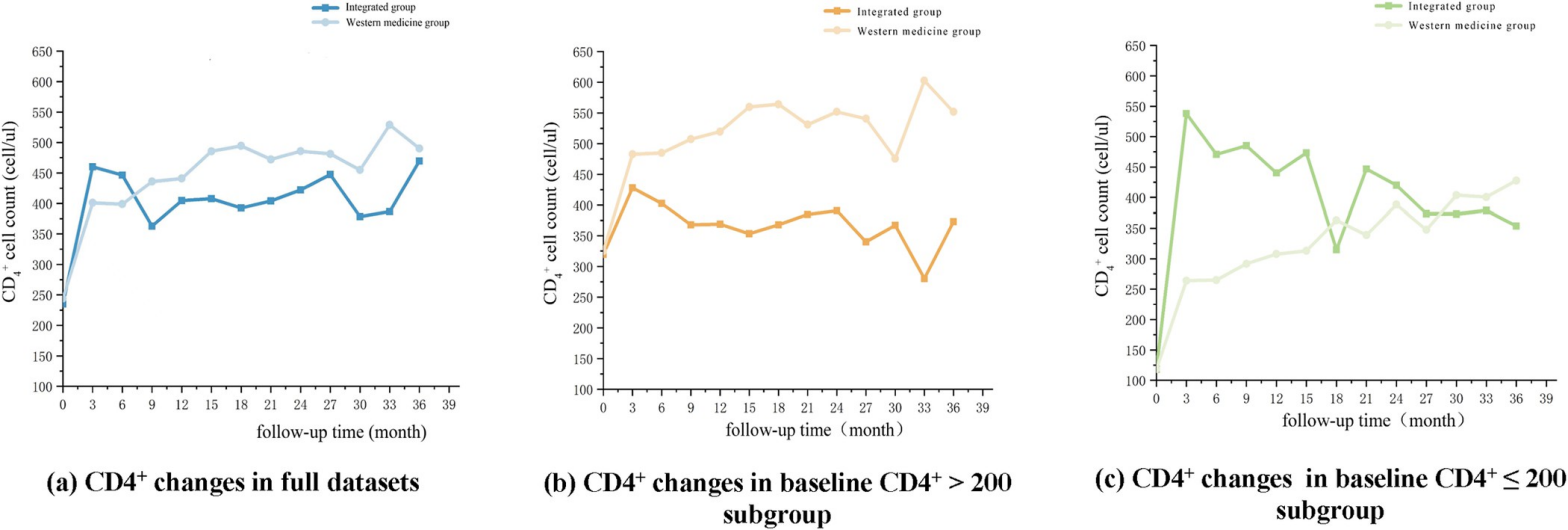
HIV/AIDS patients’ longitudinal CD4^+^ changes following PSM.

Fig 2 depict changes in CD_4_^+^ longitudinal data after PSM. Within the baseline CD4^+^ > 200 subgroup, the HAART group maintained higher CD4^+^ mean levels than the integrated group during the 36-month follow-up period. During the first 6 months of follow-up of the full dataset, CD4^+^ mean levels were higher in the integrated group than in the HAART group and after 6 months, they remained higher in the HAART group. As for the baseline CD_4_^+^ ≤ 200 subgroup, higher CD4^+^ mean levels were observed in the integrated group than in the HAART group during the 36-month follow-up period, except for the 18^th^ and 30^th^~36^th^ months. All six groups experienced rapid CD4^+^ increases during the first three months. After three months, the integrated group displayed an overall decrease, whereas the HAART group showed an accumulated increase.

### CD4^+^/CD8^+^ cell ratio

The CD4^+^/CD8^+^ ratios of patients before and after PSM are respectively presented in S4 Table and Table 3. Statistical analysis of the full dataset revealed that the CD4^+^/CD8^+^ ratio between the two groups was statistically significant at 15, 18 and 21 months, and the HAART group had a higher ratio than the integrated group. In terms of the CD_4_^+^ > 200 subgroup, the HAART group also demonstrated statistically significant higher CD4^+^/CD8^+^ ratios than the integrated group among the 12 visits of follow-up, with an exception of the 33rd month. As for the CD_4_^+^ ≤ 200 subgroup, statistically significant higher CD_4_^+^/CD_8_^+^ ratios were witnessed at the 9^th^ and 15^th^-month follow-up in the integrated group.

**Table 3:** Changes of CD_4_^+^/_8_^+^ in HIV/AIDS patients (Full dataset, CD_4_^+^ baseline > 200, baseline CD_4_^+^≤ 200) according to treatment during follow-up after PSM.

Variable	Full dataset			CD_4_^+^ baseline > 200			CD_4_^+^ baseline ≤ 200		
	Integrated group (n = 166)	HAART group (n = 166)	P values	Integrated group (n = 98)	HAART group (n = 98)	P values	Integrated group (n = 55)	HAART group (n = 55)	P values
**CD**_**4**_^**+**^**/CD**_**8**_^**+**^ **(baseline)**									
n	164	165	0.87	98	98	0.77	54	54	0.63
Median	0.24	0.25		0.34	0.33		0.16	0.14	
IQR	0.15, 0.37	0.16, 0.4		0.24, 0.47	0.24, 0.44		0.09, 0.21	0.08, 0.22	
**CD**_**4**_^**+**^**/CD**_**8**_^**+**^ **(3 months)**									
n	58	147	0.48	27	91	0.04	16	45	0.16
Median	0.44	0.41		0.37	0.51		0.28	0.24	
IQR	0.28, 0.65	0.28, 0.61		0.23, 0.66	0.37, 0.66		0.22, 0.71	0.16, 0.41	
**CD**_**4**_^**+**^**/CD**_**8**_^**+**^ **(6 months)**									
n	51	145	0.78	26	88	<0.01	10	47	0.11
Median	0.43	0.47		0.41	0.59		0.42	0.28	
IQR	0.29, 0.64	0.29, 0.66		0.24, 0.56	0.41, 0.76		0.29, 0.6	0.16, 0.42	
**CD**_**4**_^**+**^**/CD**_**8**_^**+**^ **(9 months)**									
n	48	122	0.09	34	75	<0.01	11	36	0.02
Median	0.4	0.51		0.41	0.56		0.64	0.34	
IQR	0.28, 0.57	0.33, 0.7		0.27, 0.50	0.44, 0.8		0.29, 0.96	0.16, 0.44	
**CD**_**4**_^**+**^**/CD**_**8**_^**+**^ **(12 months)**									
n	41	124	0.06	25	73	<0.01	15	37	0.31
Median	0.46	0.52		0.32	0.63		0.46	0.33	
IQR	0.27, 0.63	0.33, 0.80		0.19, 0.64	0.47, 0.96		0.3, 0.51	0.20, 0.51	
**CD**_**4**_^**+**^**/CD**_**8**_^**+**^ **(15 months)**									
n	56	91	0.01	35	58	<0.01	22	24	<0.01
Median	0.44	0.54		0.44	0.66		0.53	0.28	
IQR	0.23, 0.63	0.36, 0.77		0.2, 0.59	0.51, 1.01		0.32, 0.92	0.22, 0.41	
**CD**_**4**_^**+**^**/CD**_**8**_^**+**^ **(18 months)**									
n	45	85	0.045	20	50	<0.01	16	22	1.00
Median	0.41	0.6		0.42	0.67		0.35	0.31	
IQR	0.28, 0.65	0.32, 0.77		0.28, 0.72	0.53, 0.92		0.14, 0.70	0.21, 0.58	
**CD_4_^+^/CD_8_^+^ (21 months)**									
n	38	69	0.04	36	42	<0.01	15	20	0.44
Median	0.45	0.5		0.37	0.62		0.46	0.38	
IQR	0.24, 0.64	0.41, 0.73		0.25, 0.62	0.47, 0.82		[0.33, 0.66]	0.25, 0.46	
CD_4_^+^/CD_8_^+^ (24 months)									
n	35	52	0.09	24	28	<0.01	11	14	0.29
Median	0.45	0.60		0.31	0.80		0.44	0.28	
IQR	0.25, 0.72	0.30, 0.84		0.25, 0.65	0.62, 0.95		0.27, 0.86	0.23, 0.51	
CD_4_^+^/CD_8_^+^(27 months)									
n	47	38	0.14	34	22	<0.01	16	12	0.28
Median	0.44	0.50		0.39	0.71		0.42	0.34	
IQR	0.3, 0.66	0.35, 0.79		0.25, 0.51	0.52, 0.88		0.23, 0.65	0.23, 0.42	
CD_4_^+^/CD_8_^+^ (30 months)									
n	42	19	0.15	28	13	<0.01	17	4	0.28
Median	0.49	0.67		0.36	0.74		0.52	0.39	
IQR	0.29, 0.75	0.42, 0.85		0.26, 0.55	0.56, 0.94		0.31, 0.79	0.20, 0.55	
CD_4_^+^/CD_8_^+^ (33 months)									
n	46	13	0.84	20	7	0.10	18	4	0.59
Median	0.49	0.51		0.34	0.61		0.43	0.4	
IQR	0.26, 0.71	0.35, 0.62		0.20, 0.73	0.51, 0.78		0.36, 0.56	0.33, 0.5	
CD_4_^+^/CD_8_^+^ (36 months)									
n	22	11	0.75	17	5	0.01	6	4	1.00
Median	0.6	0.53		0.35	0.85		0.41	0.39	
IQR	0.37, 0.78	0.31, 0.85		0.24, 0.47	0.66, 0.99		0.25, 0.80	0.26, 0.53	

**Fig 3:**
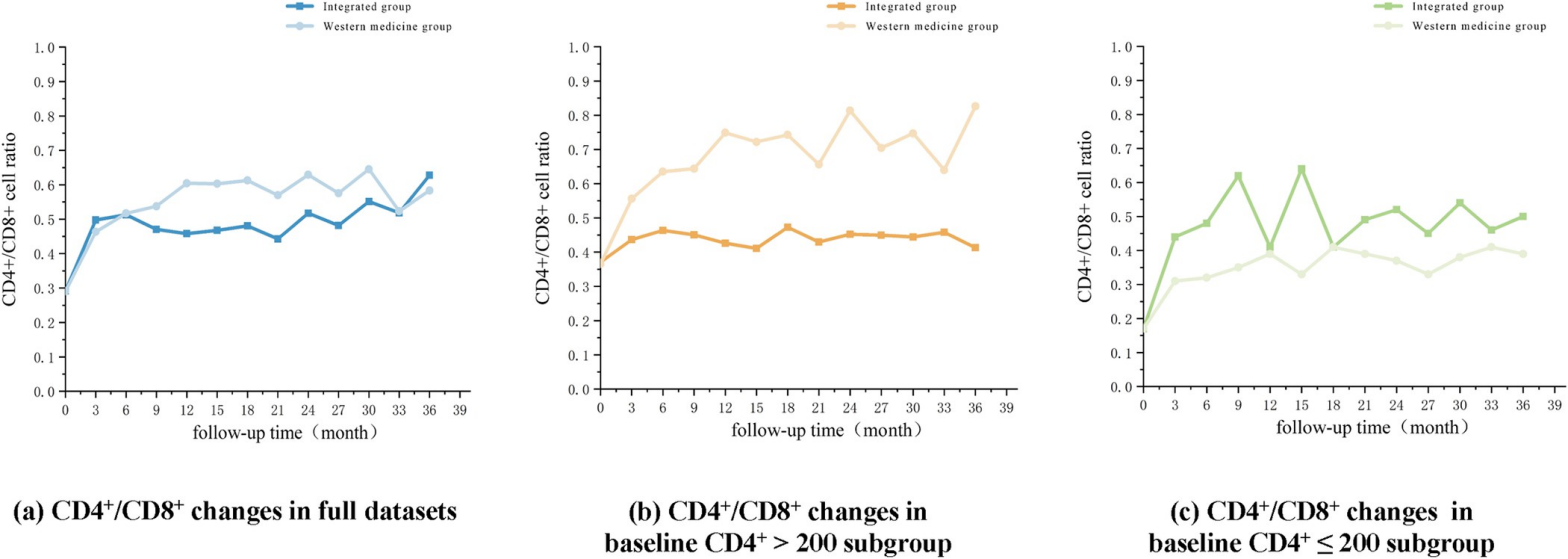
HIV/AIDS patients’ longitudinal CD4^+^/CD8^+^ changes following PSM.

The changes of CD_4_^+^/CD_8_^+^ longitudinal data after PSM were visually shown in Fig 3. For the full dataset, a greater CD_4_^+^/CD_8_^+^ mean ratio was observed in the HAART group than in the integrated group during the interval of 6 to 33 months. It was also noticed that, in the CD_4_^+^ > 200 subgroup, the ratio maintained higher levels in the HAART group throughout the study follow-up, whereas the integrated group remained at higher levels within the CD_4_^+^ ≤ 200 subgroup over the entire 36 months. All six groups experienced CD4^+^/CD8^+^ ratio increases during the first 6 months.

### Analysis results based on generalized estimating equation model

The GEE model was used to analyse the data after PSM, shown in Table 4, with the integrated group serving as the control group. On the basis of the full dataset, CD_4_^+^ (*OR* 1.119, 95%*CI* [1.018, 1.230]) and CD_4_^+^/CD_8_^+^ (*OR* 1.168, 95%*CI* [1.045, 1.305]) in the HAART group during the follow-up period were significantly higher than those in the integrated group. It was observed that in the subgroup with baseline CD4^+^ > 200, HAART group had significantly higher values of CD_4_^+^ count (*OR* 1.326, 95%*CI* [1.214, 1.449]) and CD_4_^+^/CD_8_^+^ ratio (*OR* 1.429, 95%*CI* [1.278, 1.598]) than in the integrated group, while the baseline CD4^+^ ≤ 200 subgroup showed the opposite, the integrated group revealing higher CD4^+^ (*OR* 0.825, 95%*CI* [0.694, 0.980]) and CD4^+^/CD8^+^ (*OR* 0.826, 95%*CI* [0.684, 0.997]). As far as safety outcomes are concerned, there were no statistically significant differences between the integrated and the HAART groups in the three datasets in terms of BUN, ALT and AST. Cr level was found significantly higher in the HAART group in all three datasets.

**Table 4:** Analysis of longitudinal data for HIV/AIDS patients based on the generalized estimating equation model.

Variable	Full dataset		CD_4_^+^ baseline > 200		CD_4_^+^ baseline ≤ 200	
	*OR*	95% *CI*	*OR*	95% *CI*	*OR*	95% *CI*
CD_4_^+^	1.119	[1.018, 1.230]	1.326	[1.214, 1.449]	0.825	[0.694, 0.980]
CD_4_^+^/CD_8_^+^	1.168	[1.045, 1.305]	1.429	[1.278, 1.598]	0.826	[0.684, 0.997]
Cr	1.166	[1.112, 1.222]	1.187	[1.113, 1.266]	1.204	[1.133, 1.280]
BUN	0.969	[0.840, 1.118]	0.963	[0.879, 1.054]	0.790	[0.621, 1.005]
ALT	0.934	[0.789, 1.105]	1.013	[0.802, 1.280]	0.979	[0.770, 1.246]
AST	0.937	[0.845, 1.039]	1.002	[0.872, 1.151]	1.011	[0.867, 1.179]

Notes: Cr refers to creatinine; BUN refers to blood urea nitrogen; ALT refers to alanine transaminase; AST refers to aspartate aminotransferase

## Discussion

We analyzed the real-world longitudinal data to explore the effectiveness and safety of CHM for patients with HIV/AIDS. The patients were followed up for 36 months. A 1:1 PSM was performed to balance the baseline and eliminate confounding factors. The longitudinal data were analyzed using a GEE model. A final sample of 629 patients, 455 and 174 in the integrated group and HAART group respectively, were obtained from the full dataset. Following PSM, 166 pairs from the full dataset were matched successfully, with 98 pairs in the baseline CD_4_^+^ > 200 subgroup, and 55 pairs in the baseline CD_4_^+^ ≤ 200 subgroup. In the full dataset, HAART group achieved higher CD4^+^ count (*OR* 1.119, 95%*CI* [1.018, 1.230]) and CD4^+^/CD_8_^+^ ratio (*OR* 1.168, 95%*CI* [1.045, 1.305]) than the integrated group. Higher level of CD4^+^ count (*OR* 1.326, 95%*CI* [1.214, 1.449]) and CD4^+^/CD8^+^ ratio (*OR* 1.429, 95%*CI* [1.278, 1.598]) were also observed in the CD4^+^ > 200 subgroup. For the CD_4_^+^ ≤ 200 subgroup, the CD4^+^ (*OR* 0.825, 95%*CI* [0.694, 0.980]) and CD4^+^/CD_8_^+^ (*OR* 0.826, 95%*CI* [0.684, 0.997] of the integrated group were higher than those of the HAART group. The safety outcomes showed that there were no significant differences in BUN, ALT and AST levels between the groups but Cr showed significantly higher levels in HAART groups of all three datasets.

In spite of the high external validity of real-world data, there are a number of confounding factors that can make causal inferences less accurate. To improve the internal validity of inferences, matching methods are often applied to real-world data. To improve the accuracy of the results of this study, we use PSM to determine the baseline and to balance both the internal and external validity of real-world data. Meanwhile, using CD4^+^ as the outcome variable, we converted longitudinal continuous variables into dichotomous variables, dividing them into dominant and inferior populations. As covariates for PSM, gender, age, baseline CD_4_^+^ and CD_4_^+^/ CD_8_^+^ were assessed based on the results of the logistic regression analyses. For a more in-depth analysis, we also divided the CD4^+^ baseline into two subgroups, baseline CD_4_^+^ > 200 and baseline CD_4_^+^ ≤ 200 subgroups. In the GEE model, repeated measures are fully taken into account. The GEE is also capable of analyzing a variety of types of outcome variables, as well as processing data with missing data with different observation times and time intervals for observed objects.

The limitation of this study mainly lies in the high rate of lost follow-up of data. Although an appropriate analytical model is adopted for processing, the high rate of lost follow-up will have a certain impact on the accuracy of the results. In addition, PSM can only make equilibrium adjustments for known and measurable covariables, but cannot control the effect of unknown or unmeasured covariables on the outcome effect. Only when all covariables are known and measurable can the unbiased estimation of outcome effects be truly realized.

The results of the subgroup (baseline CD4^+^ > 200) showed that the HAART group was superior to the integrated group to improve patients’ immune function, whereas the full dataset and the baseline CD4^+^ ≤ 200 subgroup revealed that the integrated group was more beneficial. The result is consistent with a previous cohort study we conducted without PSM, showing that the CD4^+^ counts of the integrated group remained significantly lower than those of the HAART group in the first 3 years [25]. However, the results of the two subgroups were in opposite directions, showing qualitative differences, which is relatively rare. Chance alone is unlikely to account for significant subgroup effects, which may not be genuine [26]. Data from a single study can only generate hypotheses about differences between subgroups. To enhance credibility, it is essential to replicate such findings through repeated studies.

## Conclusion

The results of the study show that after three years of treatment, the difference between CHMs combined with antiviral therapy and the use of antiviral therapy alone in improving the immune function of HIV infection and AIDS patients has no clinical significance. The results of the two subgroups are in opposite directions, and chance does not explain the apparent subgroup effect. A study with a larger sample size and longer follow-up period is warranted in order to increase study credibility.

## Supporting information

S1 Table Single-factor comparison of characteristics between dominant and inferior patients. (DOCX)

S2 Table Results of logistic regression analysis. (DOCX)

S3 Table CD4^+^ of HIV/ AIDS patients before PSM (full dataset, CD4^+^ > 200, CD4^+^≤ 200) grouped by treatment methods. (DOCX)

S4 Table CD_4_^+^/CD_8_^+^ of patients before PSM during follow-up (full dataset, baseline CD_4_^+^ > 200, baseline CD_4_^+^≤ 200) grouped by treatment methods. (DOCX)

## Abbreviation

AIDS: Acquired immunodeficiency syndrome
ALT: Alanine transaminase
AST: Aspartate aminotransferase
BUN: Blood urea nitrogen
CHM: Chinese herbal medicine
Cr: Creatinine
FIs: Fusion inhibitors
GEE: Generalized estimating equation
HAART: Highly active antiretroviral therapy
HIV: Human immunodeficiency virus
INSTIs: Integrase inhibitors
NFCHMP: National Free CHM HIV/AIDS Treatment Program
NNRTIs: Non-nucleoside reverse transcriptase inhibitors
NRTIs: Nucleoside reverse transcriptase inhibitors
PIs: Protease inhibitors
PSM: Propensity score matching
RCT: Randomized controlled trial
RWS: Real-world study
SATCHM: State Administration of Traditional Chinese Medicine
TCM: Traditional Chinese medicine
UNAIDS: Joint United Nations Programme on HIV/AIDS
