# Supplementary material for: Chinese herbal medicine for patients living with HIV in Guangxi province, China: A propensity score matching analysis of real-world data
Source: PLoS One. 2024 Sep 6;19(9):e0304332. doi: 10.1371/journal.pone.0304332 (PMC11379241; doi:10.1371/journal.pone.0304332)
Supplement: S1 Table — (DOCX) [file pone.0304332.s001.docx]

**S1 Table. Single-factor comparison of characteristics between dominant and inferior patients.**

| **Variables** | **Immune function** | | ***P* value** |
| --- | --- | --- | --- |
|  | **Dominant patients (*n*=299)** | **Inferior patients (*n*=330)** |  |
| **Gender—*n* (%)** | | | |
| *n* | 299 | 330 | 0.18 |
| Male | 216(72.24%) | 222(67.27%) |  |
| Female | 83(27.76%) | 108(32.73%) |  |
| **Age—year** | | | |
| *n* | 299 | 330 | 0.07 |
| Median | 41 | 45 |  |
| 95%*CI* | [17.13, 70.59] | [18.50, 73.42] |  |
| **Marital status—*n* (%)** | | | |
| *n* | 299 | 330 | 0.67 |
| Unmarried | 52(17.39%) | 48(14.55%) |  |
| Married | 191(63.88%) | 220(66.67%) |  |
| Divorced | 18(6.02%) | 16(4.84%) |  |
| Death of a spouse | 38(12.71%) | 46(13.94%) |  |
| **Possible route of infection—*n* (%)** | | | |
| *n* | 299 | 330 | 0.60 |
| Drug-taking | 30(10.03%) | 33(10.00%) |  |
| Sexual contact | 252(84.28%) | 276(83.64%) |  |
| Mother-to-child transmission | 8(2.68%) | 12(3.64%) |  |
| Paid blood | 0(0) | 2(0.60%) |  |
| Unknown | 9(3.01%) | 7(2.12%) |  |
| **CD_4_^+^—cell/ul** | | | |
| *n* | 299 | 330 | <0.01 |
| Median | 189 | 262 |  |
| IQR | 114, 259 | 187.5, 372 |  |
| **CD_8_^+^—cell/ul** | | | |
| *n* | 297 | 327 | 0.04 |
| Median | 767 | 834 |  |
| IQR | 521.5, 1044.5 | 559, 1182 |  |
| **CD_4_^+^/CD_8_^+^** | | | |
| *n* | 297 | 327 | <0.01 |
| Median | 0.24 | 0.33 |  |
| IQR | 0.14, 0.39 | 0.22, 0.5 |  |
| **Cr—μmol/L** | | | |
| *n* | 262 | 301 | 0.99 |
| Median | 74.95 | 74 |  |
| IQR | 64.08, 88 | 63.2, 88 |  |
| **BUN—mmol/L** | | | |
| *n* | 268 | 310 | 0.22 |
| Median | 4.46 | 4.70 |  |
| IQR | 3.59, 5.46 | 3.62, 5.65 |  |
| **ALT—U/L** | | | |
| *n* | 289 | 317 | 0.66 |
| Median | 21 | 21 |  |
| IQR | 15, 33 | 15, 34 |  |
| **AST—U/L** | | | |
| *n* | 286 | 316 | 0.51 |
| Median | 24.75 | 24 |  |
| IQR | 20, 34.48 | 19.23, 34.75 |  |
