# Supplementary material for: Chinese herbal medicine for patients living with HIV in Guangxi province, China: A propensity score matching analysis of real-world data
Source: PLoS One. 2024 Sep 6;19(9):e0304332. doi: 10.1371/journal.pone.0304332 (PMC11379241; doi:10.1371/journal.pone.0304332)
Supplement: S2 Table — (DOCX) [file pone.0304332.s002.docx]

**S2 Table. Results of logistic regression analysis.**

| **Independent variables** | ***OR*** | **95%*CI*** |
| --- | --- | --- |
| Age | 0.983 | [0.970, 0.996] |
| CD_4_+ | 0.994 | [0.993, 0.996] |
