# Supplementary material for: Chinese herbal medicine for patients living with HIV in Guangxi province, China: A propensity score matching analysis of real-world data
Source: PLoS One. 2024 Sep 6;19(9):e0304332. doi: 10.1371/journal.pone.0304332 (PMC11379241; doi:10.1371/journal.pone.0304332)
Supplement: S3 Table — (DOCX) [file pone.0304332.s003.docx]

**S3 Table. CD_4_^+^ of HIV/ AIDS patients before PSM (full dataset, CD_4_^+^ > 200, CD_4_^+^≤ 200) grouped by treatment methods.**

| **Variables** | **Full dataset** | | | **Baseline CD_4_^+^ >200** | | | **Baseline CD_4_^+^ < 200** | | |
| --- | --- | --- | --- | --- | --- | --- | --- | --- | --- |
|  | **Integrated group (n=455)** | **HAART group**  **(n=174)** | **P value** | **Integrated group (n=260)** | **HAART group**  **(n=110)** | **P value** | **Integrated group (n=195)** | **HAART group**  **(n=64)** | **P value** |
| **CD_4_^+^(baseline)—cell/ul** | | | | | | | | | |
| n | 455 | 174 | 0.23 | 260 | 110 | 0.42 | 195 | 64 | 0.18 |
| Median | 227 | 243.5 |  | 282. | 312 |  | 133 | 126 |  |
| IQR | 148.00, 295.00 | 154.75, 344.25 |  | 240, 388.25 | 248.75, 372.25 |  | 92, 172 | 66.5, 170.75 |  |
| **CD_4_^+^(3 months)—cell/ul** | | | | | | | | | |
| n | 153 | 153 | 0.65 | 87 | 101 | <0.01 | 66 | 52 | <0.01 |
| Median | 378 | 392 |  | 371 | 457 |  | 414 | 240 |  |
| IQR | 250.50, 503.00 | 255.5, 539 |  | 244, 467 | 352, 591 |  | 277.25, 568 | 168.5, 331.25 |  |
| **CD_4_^+^(6 months)—cell/ul** | | | | | | | | | |
| n | 145 | 153 | 0.92 | 78 | 99 | <0.01 | 67 | 54 | <0.01 |
| Median | 385 | 392 |  | 385.5 | 452 |  | 385 | 255 |  |
| IQR | 249.00, 536.00 | 279.5, 511 |  | 224.75, 463.5 | 371, 561 |  | 275, 608 | 173.75, 327 |  |
| **CD_4_^+^(9 months)—cell/ul** | | | | | | | | | |
| n | 158 | 127 | 0.06 | 93 | 84 | <0.01 | 65 | 43 | <0.01 |
| Median | 364.50 | 416 |  | 339 | 460.5 |  | 408 | 254 |  |
| IQR | 242.50, 504.00 | 296, 535 |  | 224, 481.5 | 367.5, 581.75 |  | 252, 565 | 201, 379 |  |
| **CD_4_^+^(12 months)—cell/ul** | | | | | | | | | |
| n | 135 | 131 | 0.11 | 85 | 85 | <0.01 | 50 | 46 | <0.01 |
| Median | 372 | 415 |  | 378 | 467 |  | 371.5 | 271.5 |  |
| IQR | 226.00, 522.00 | 275, 518 |  | 213.5, 505.5 | 388.5, 588 |  | 239.5, 561.75 | 209.75, 357.75 |  |
| **CD_4_^+^(15 months)—cell/ul** | | | | | | | | | |
| n | 163 | 93 | <0.01 | 81 | 66 | <0.01 | 82 | 27 | <0.01 |
| Median | 375 | 453 |  | 330 | 499 |  | 417.5 | 276 |  |
| IQR | 235.00, 542.00 | 324, 576.5 |  | 193.5, 525.5 | 437.25, 644.25 |  | 283.75, 569.5 | 214, 356 |  |
| **CD_4_^+^(18 months)—cell/ul** | | | | | | | | | |
| n | 127 | 89 | <0.01 | 70 | 59 | <0.01 | 57 | 30 | 0.78 |
| Median | 342 | 458 |  | 366 | 495 |  | 333 | 338.5 |  |
| IQR | 209.00, 514.00 | 348, 592 |  | 211.25, 509.5 | 423, 665 |  | 203.5, 516 | 257.75, 450.5 |  |
| **CD_4_^+^(21 months)—cell/ul** | | | | | | | | | |
| n | 129 | 71 | <0.01 | 78 | 49 | <0.01 | 51 | 22 | 0.33 |
| Median | 367 | 457 |  | 371 | 520 |  | 359 | 325 |  |
| IQR | 245.50, 510.50 | 344, 542 |  | 266.5, 488.75 | 427, 606.5 |  | 236, 596 | 293, 408.75 |  |
| **CD_4_^+^(24 months)—cell/ul** | | | | | | | | | |
| n | 112 | 54 | 0.03 | 62 | 34 | <0.01 | 50 | 20 | 0.52 |
| Median | 369 | 465 |  | 376 | 556.5 |  | 366 | 322.5 |  |
| IQR | 236.25, 537.75 | 302.75, 599.5 |  | 233.25, 491.25 | 353, 645.25 |  | 241.75, 624.25 | 244.25, 492 |  |
| **CD_4_^+^(27 months)—cell/ul** | | | | | | | | | |
| n | 140 | 39 | <0.01 | 82 | 25 | <0.01 | 58 | 14 | 0.74 |
| Median | 342.5 | 446 |  | 341 | 528 |  | 361 | 353.5 |  |
| IQR | 216.25, 506.75 | 357, 604 |  | 215.5, 498 | 445, 664 |  | 229, 518.75 | 269.25, 406 |  |
| **CD_4_^+^(30 months)—cell/ul** | | | | | | | | | |
| n | 123 | 20 | 0.01 | 65 | 14 | <0.01 | 58 | 6 | 0.73 |
| Median | 364 | 431.5 |  | 358 | 456 |  | 366.5 | 393.5 |  |
| IQR | 274.00, 450.00 | 369.75, 549 |  | 282, 447.5 | 389.25, 557.75 |  | 272, 499.25 | 272.75, 517.75 |  |
| **CD_4_^+^(33 months)—cell/ul** | | | | | | | | | |
| n | 125 | 13 | <0.01 | 62 | 8 | 0.01 | 63 | 5 | 0.30 |
| Median | 327 | 451 |  | 329.5 | 608 |  | 327 | 396 |  |
| IQR | 200.00, 487.00 | 383.5, 643 |  | 200.75, 505.75 | 414.75, 805.5 |  | 195, 486 | 366.5, 435 |  |
| **CD_4_^+^(36 months)—cell/ul** | | | | | | | | | |
| n | 79 | 12 | 0.18 | 49 | 6 | 0.19 | 30 | 6 | 0.42 |
| Median | 387 | 462 |  | 419 | 488 |  | 374.5 | 423.5 |  |
| IQR | 244.00, 527.00 | 409, 499.25 |  | 241.5, 561 | 412.25, 701.5 |  | 258.75, 509 | 394.75, 467 |  |
