# Supplementary material for: Chinese herbal medicine for patients living with HIV in Guangxi province, China: A propensity score matching analysis of real-world data
Source: PLoS One. 2024 Sep 6;19(9):e0304332. doi: 10.1371/journal.pone.0304332 (PMC11379241; doi:10.1371/journal.pone.0304332)
Supplement: S4 Table — (DOCX) [file pone.0304332.s004.docx]

**S4 Table. CD_4_^+^/CD_8_^+^ of patients before PSM during follow-up (full dataset, baseline CD_4_^+^ > 200, baseline CD_4_^+^≤ 200) grouped by treatment methods.**

| **Variables** | **Full dataset** | | | **Baseline CD_4_^+^ > 200** | | | **Baseline CD_4_^+^ < 200** | | |
| --- | --- | --- | --- | --- | --- | --- | --- | --- | --- |
|  | **Integrated group (n=455)** | **HAART group**  **(n=174)** | **P value** | **Integrated group (n=260)** | **HAART group**  **(n=110)** | **P value** | **Integrated group (n=195)** | **HAART group**  **(n=64)** | **P value** |
| **CD_4_^+^/CD_8_^+^(baseline)—cell/ul** | | | | | | | | | |
| n | 452 | 172 | <0.01 | 259 | 109 | <0.01 | 193 | 63 | <0.01 |
| Median | 0.3 | 0.25 |  | 0.41 | 0.33 |  | 0.19 | 0.12 |  |
| IQR | 0.19, 0.47 | 0.15, 0.39 |  | 0.27, 0.57 | 0.23 0.45 |  | 0.12, 0.3 | 0.08, 0.22 |  |
| **CD_4_^+^/CD_8_^+^(3 months)—cell/ul** | | | | | | | | | |
| n | 153 | 153 | 0.39 | 87 | 101 | 0.13 | 66 | 52 | <0.01 |
| Median | 0.43 | 0.41 |  | 0.44 | 0.5 |  | 0.4 | 0.24 |  |
| IQR | 0.26, 0.64 | 0.28, 0.61 |  | 0.26, 0.66 | 0.36, 0.67 |  | 0.26, 0.61 | 0.14, 0.41 |  |
| **CD_4_^+^/CD_8_^+^(6 months)—cell/ul** | | | | | | | | | |
| n | 145 | 152 | 0.88 | 78 | 98 | <0.01 | 67 | 54 | <0.01 |
| Median | 0.45 | 0.47 |  | 0.44 | 0.59 |  | 0.49 | 0.27 |  |
| IQR | 0.30, 0.63 | 0.29, 0.66 |  | 0.27, 0.57 | 0.40, 0.76 |  | 0.34, 0.64 | 0.15, 0.42 |  |
| **CD_4_^+^/CD_8_^+^(9 months)—cell/ul** | | | | | | | | | |
| n | 157 | 126 | 0.13 | 93 | 84 | <0.01 | 64 | 42 | <0.01 |
| Median | 0.42 | 0.49 |  | 0.37 | 0.56 |  | 0.49 | 0.29 |  |
| IQR | 0.29, 0.63 | 0.32, 0.69 |  | 0.27, 0.57 | 0.43, 0.79 |  | 0.33, 0.69 | 0.16, 0.41 |  |
| **CD_4_^+^/CD_8_^+^(12 months)—cell/ul** | | | | | | | | | |
| n | 135 | 129 | 0.02 | 85 | 84 | <0.01 | 50 | 45 | <0.01 |
| Median | 0.44 | 0.51 |  | 0.38 | 0.62 |  | 0.46 | 0.31 |  |
| IQR | 0.27, 0.63 | 0.33, 0.79 |  | 0.25, 0.65 | 0.44, 0.94 |  | 0.34, 0.53 | 0.18, 0.49 |  |
| **CD_4_^+^/CD_8_^+^(15 months)—cell/ul** | | | | | | | | | |
| n | 161 | 93 | <0.01 | 81 | 66 | <0.01 | 80 | 27 | <0.01 |
| Median | 0.44 | 0.53 |  | 0.33 | 0.65 |  | 0.49 | 0.27 |  |
| IQR | 0.25, 0.60 | 0.36, 0.77 |  | 0.23, 0.57 | 0.51, 0.90 |  | 0.30, 0.68 | 0.21, 0.39 |  |
| **CD_4_^+^/CD_8_^+^(18 months)—cell/ul** | | | | | | | | | |
| n | 127 | 89 | <0.01 | 70 | 59 | <0.01 | 57 | 30 | 0.06 |
| Median | 0.41 | 0.57 |  | 0.38 | 0.66 |  | 0.41 | 0.25 |  |
| IQR | 0.27, 0.62 | 0.3, 0.77 |  | 0.27, 0.64 | 0.51, 0.92 |  | 0.28, 0.63 | 0.20, 0.54 |  |
| **CD_4_^+^/CD_8_^+^(21 months)—cell/ul** | | | | | | | | | |
| n | 128 | 71 | <0.01 | 77 | 49 | <0.01 | 51 | 22 | 0.13 |
| Median | 0.39 | 0.49 |  | 0.34 | 0.62 |  | 0.46 | 0.38 |  |
| IQR | 0.23, 0.65 | 0.4, 0.72 |  | 0.23, 0.56 | 0.45, 0.8 |  | 0.27, 0.71 | 0.24, 0.46 |  |
| **CD_4_^+^/CD_8_^+^(24 months)—cell/ul** | | | | | | | | | |
| n | 112 | 54 | 0.03 | 62 | 34 | <0.01 | 52 | 20 | 0.02 |
| Median | 0.42 | 0.58 |  | 0.34 | 0.79 |  | 0.48 | 0.26 |  |
| IQR | 0.26, 0.71 | 0.30, 0.83 |  | 0.25, 0.70 | 0.57, 0.94 |  | 0.29, 0.71 | 0.22, 0.49 |  |
| **CD_4_^+^/CD_8_^+^(27 months)—cell/ul** | | | | | | | | | |
| n | 139 | 39 | 0.02 | 82 | 25 | <0.01 | 57 | 14 | 0.29 |
| Median | 0.39 | 0.5 |  | 0.41 | 0.71 |  | 0.38 | 0.34 |  |
| IQR | 0.26, 0.63 | 0.36, 0.78 |  | 0.26, 0.66 | 0.49, 0.89 |  | 0.26, 0.62 | 0.24, 0.44 |  |
| **CD_4_^+^/CD_8_^+^(30 months)—cell/ul** | | | | | | | | | |
| n | 123 | 19 | 0.02 | 65 | 14 | <0.01 | 58 | 5 | 0.42 |
| Median | 0.41 | 0.67 |  | 0.41 | 0.75 |  | 0.40 | 0.29 |  |
| IQR | 0.28, 0.67 | 0.42, 0.85 |  | 0.28, 0.68 | 0.61, 0.93 |  | 0.29, 0.67 | 0.22, 0.54 |  |
| **CD_4_^+^/CD_8_^+^(33 months)—cell/ul** | | | | | | | | | |
| n | 124 | 13 | 0.31 | 62 | 8 | 0.32 | 62 | 5 | 0.81 |
| Median | 0.44 | 0.51 |  | 0.48 | 0.59 |  | 0.43 | 0.33 |  |
| IQR | 0.25, 0.65 | 0.35, 0.62 |  | 0.25, 0.75 | 0.44, 0.74 |  | 0.24, 0.54 | 0.325, 0.49 |  |
| **CD_4_^+^/CD_8_^+^(36 months)—cell/ul** | | | | | | | | | |
| n | 78 | 11 | 0.31 | 48 | 5 | 0.03 | 30 | 6 | 0.39 |
| Median | 0.46 | 0.53 |  | 0.46 | 0.85 |  | 0.47 | 0.36 |  |
| IQR | 0.29, 0.64 | 0.31, 0.85 |  | 0.28, 0.71 | 0.66, 0.99 |  | 0.33, 0.60 | 0.26, 0.52 |  |
